# Disseminated zoster in an adult patient with extensive burns: a case report

**DOI:** 10.1186/s12985-019-1179-8

**Published:** 2019-05-23

**Authors:** Yoshitaka Kubota, Kentaro Kosaka, Toshinori Hokazono, Yoshihisa Yamaji, Takafumi Tezuka, Shinsuke Akita, Motone Kuriyama, Nobuyuki Mitsukawa

**Affiliations:** 1 Department of Plastic Surgery, Chiba University, 1-8-1, Inohana, Chuo-ku, Chiba-city, Chiba #260-8677 Japan; 2 Department of Plastic Surgery, Imakiire General Hospital, 4-16, Shimotatsuo-cho, Kagoshima-city, Kagoshima #892-0852 Japan

**Keywords:** Burn, Varicella zoster virus, Herpes simplex virus, Reactivation, Air-borne infection, Hemodialysis

## Abstract

**Background:**

Shingles (localized zoster) and disseminated zoster are caused by the reactivation of latent varicella zoster virus (VZV). Reactivation of VZV is related to impaired cell-mediated immunity. Extensive burns affecting a patient result in burn-related immunosuppression and cytokine storm. Despite immunosuppression in burn patients, the reactivation of VZV is extremely rare, whereas eczema herpeticum, caused by reactivation of latent herpes simplex virus (HSV), is common. We have found only 1 published case of VZV reactivation during burn treatment in the literature.

**Case presentation:**

A 51-year-old man was burned in a fire, which affected 60% of his total body surface area (TBSA), and also received inhalation injury (day 0). Despite fluid resuscitation, he showed persistent renal failure. Continuous hemodialysis and filtration (CHDF) combined with polymyxin B-immobilized fiber column direct hemoperfusion (PMX-DHP) therapy was used for cytokine modulation. Autologous and allogeneic skin grafting was performed. On day 15, multiple-drug-resistant *Pseudomonas aeruginosa* (MDRP) was detected from a blood specimen, and the patient developed multiple organ failure (MOF). On day 31, compact aggregations of small vesicles appeared on the intact skin of his left knee and left buttock. The vesicles were located within the 4th lumbar (L4) spinal dermatome. From day 32 to day 34, similar new vesicles arose on his intact skin and epithelializing skin-graft donor sites. We diagnosed disseminated zoster, based on the patient’s age, the characteristic occurrence of the initial vesicles within a limited area of intact skin in the left L4 dermatome, and a positive Tzank smear. Serologic testing on day 36 showed a high level of anti-VZV immunoglobulin (Ig)G with low levels of anti-VZV IgM, anti-HSV IgG, and anti-HSV IgM. The patient was isolated in a negative-pressure room to avoid air-borne spread of VZV. On day 52, the patient died.

**Conclusions:**

To the best of our knowledge, our patient is the second case of reactivation of VZV during burn treatment. It is unclear why reactivation of VZV is rare in patients with burn-related immunosuppression, whereas HSV reactivation is common. Cytokine modulation throughout the treatment period using CHDF combined with PMX-DHP might have been related to the rare reactivation of VZV in our patient. Our case provides an additional information on the relationship between the immune status of a patient with extensive burns and reactivation of latent VZV or HSV.

## Background

Disseminated zoster, caused by the reactivation of latent varicella zoster virus (VZV), is sometimes seen in patients with immunocompromised status, such as post-organ transplantation, lymphoma, and acquired immunodeficiency syndrome (AIDS) [1]. Extensive burns affecting a patient result in burn-related immunosuppression and cytokine storm. Despite immunosuppression in burn patients, the reactivation of VZV is extremely rare, whereas eczema herpeticum, also known as Kaposi’s varicelliform eruption (KVE), caused by reactivation of latent herpes simplex virus (HSV) is common. To the best of our knowledge, only 1 case of VZV reactivation during burn treatment can be found in the literature [2]. Herein, we report a burn patient who developed disseminated zoster as a complication of burn treatment, which required airborne infection isolation.

## Case presentation

A 51-year-old unemployed Asian man sustained a thermal burn affecting 60% of his total body surface area (TBSA) in a gasoline fire. He arrived at our hospital 4 h after injury (day 0). Upon initial examination, he was in shock and found to have inhalation injury. He had third degree burns on his face, head, neck, chest, back, bilateral upper extremities, abdomen, and bilateral thighs. Fluid resuscitation and artificial ventilation with tracheal intubation were started. According to his family, he did not have any obvious comorbidities. The patient’s history of VZV vaccination and VZV infection were unknown. Despite fluid resuscitation and catecholamine support, he had persistent oliguria and hypotension. Continuous hemodialysis and filtration (CHDF) combined with polymyxin B-immobilized fiber column direct hemoperfusion (PMX-DHP) was started on day 1 to treat his renal failure, reduce cytokine storm, and remove endotoxins in an attempt to prevent development of multiple organ failure (MOF). Multiple eschar debridements and autologous and allogeneic skin grafting were performed. Almost complete escharectomy was achieved by day 13. However, throughout the treatment period, the patient’s general condition remained critical. He could not be weaned from artificial ventilation and CHDF, and he continuously needed catecholamine circulatory support. On day 10, he developed cardiac arrest that was treated by cardiac massage and intravenous adrenaline, with return of spontaneous circulation (ROSC).

**Fig. 1:**
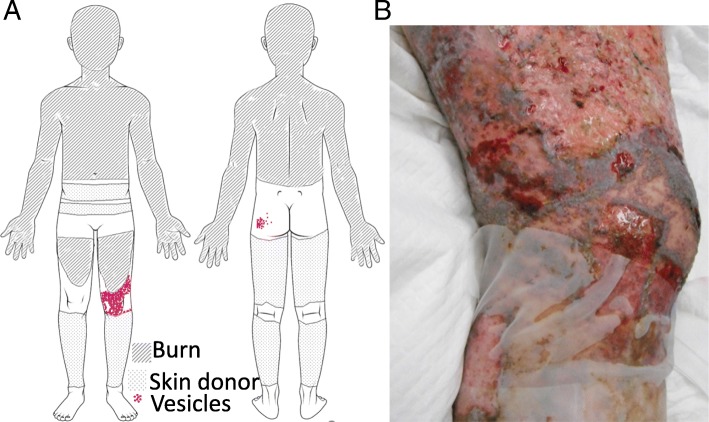
Vesicles at onset of eruption on day 31. **a** Distribution of vesicles on day 31. Aggregated vesicles were seen on intact skin of the left knee and left buttock along in the fourth lumbar nerve root (L4) dermatome. **b** Photograph of the left knee on day 31. Aggregated vesicles on the intact skin of the left knee

**Fig. 2:**
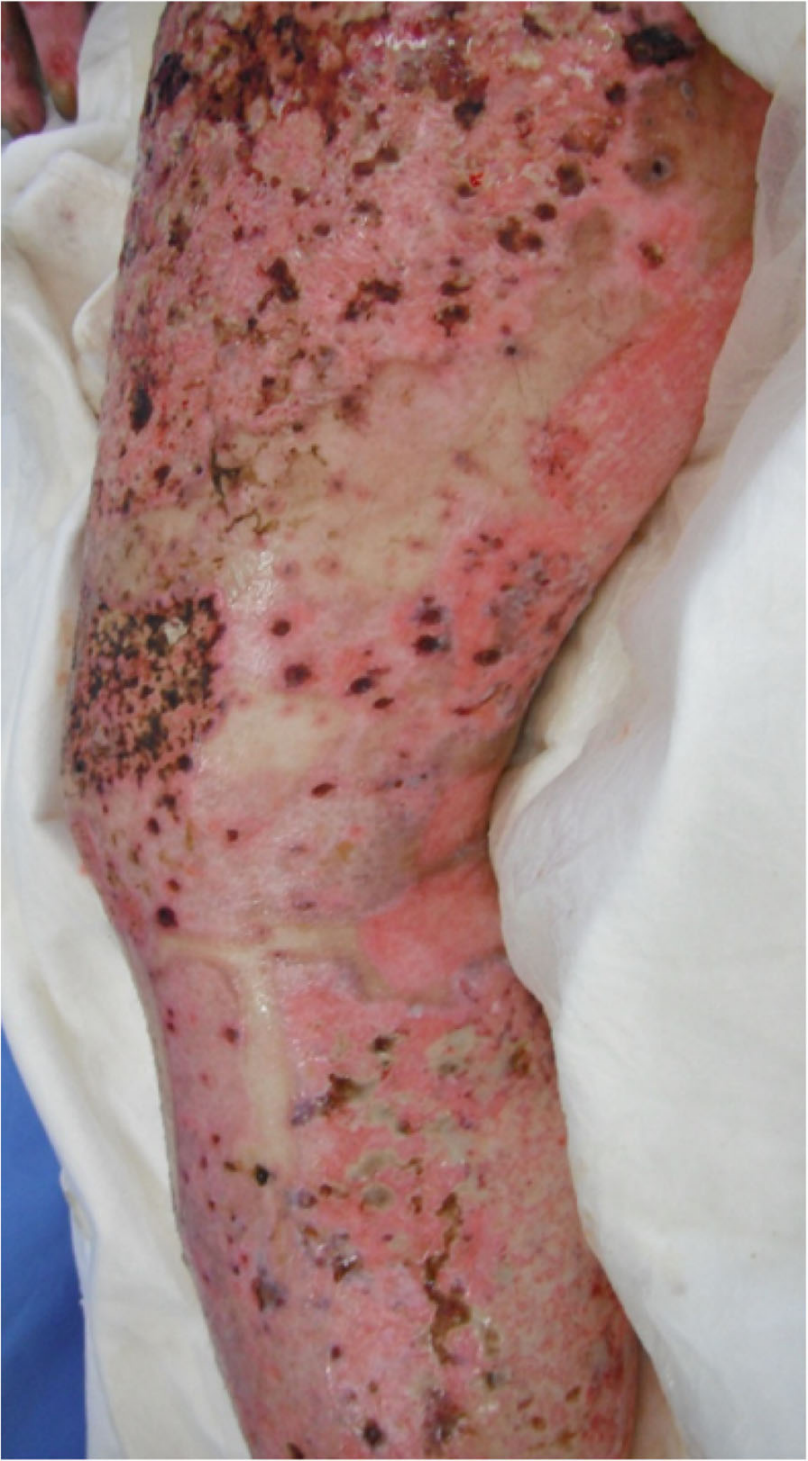
Vesicles on day 48. Ulcers resulting from unhealed vesicles on right thigh, knee, and leg

Engraftment of skin grafts and epithelialization of the skin donor sites were poor, and on day 15, multiple-drug-resistant *Pseudomonas aeruginosa* (MDRP) was detected in specimens from his burn wound and blood. His burn sepsis was refractory to treatment, and he developed multiple organ failure (MOF). On day 31, compact aggregations of small vesicles appeared on the intact skin of his left knee and left buttock. The vesicles were located within the 4th lumbar (L4) spinal dermatome (Fig. 1). From day 32 to day 34, similar new vesicles arose on his intact skin and epithelializing split-thickness skin graft harvested sites on both legs, both knees, entire abdomen, both buttocks, and entire face and neck. The vesicles ruptured sequentially and became multiple skin ulcers. On day 34, the patient was evaluated by dermatologists, and was diagnosed with disseminated zoster caused by reactivation of latent varicella zoster virus (VZV), based on the patient’s age, characteristic pattern of aggregations of vesicles initially limited to the left L4 dermatome, and positive Tzank smear (Giemsa-stained vesicular content positive for multinucleated giant cells). Because disseminated zoster can be transmitted via an airborne route, the patient was isolated in a negative-pressure room to avoid airborne spread of VZV. On day 36, the patient’s serum levels of anti-VZV immunoglobulin (Ig) G, anti-VZV IgM, anti-HSV IgG, and anti-HSV IgM antibodies were measured by enzyme-linked immunosorbent assay (ELISA) kits (Denka Seiken, Tokyo, Japan). The antigen in the measuring kits of anti-VZV IgG and anti-VZV IgM is isolated from Vero cells infected with VZV HS strain. The antigen in the measuring kits of anti-HSV IgG and anti-HSV IgM is isolated from RD cells infected with HSV H-S1 strain. The antibody titers calculated from a standard curve using ELISA OD values. The results showed a high anti-VZV IgG antibody titer of 128 (> 4.0 is defined as positive according to the manufacturer’s insert) and low anti-VZV IgM antibody titer of 0.46 (< 0.8 defined as negative by the manufacturer’s insert). The anti-HSV IgG antibody titer was indeterminant at 2.5 (> 4, positive; < 2.0, negative; 2.0–3.9, indeterminant by the manufacturer’s insert), and the anti-HSV IgM antibody titer was negative at 0.47 (< 0.8 defined as negative by the manufacturer’s insert). Intravenous acyclovir, 500 mg daily, was started. Despite intensive care and acyclovir for 14 days, the patient’s general condition worsened. On day 48, most vesicles had not healed and had become skin ulcers (Fig. 2), and not much skin was available skin for skin grafts. Therefore, treating the burn was impossible. On day 52, the patient died of multiple organ failure. No cases of VZV infection occurred in other ICU patients or ICU personnel during the treatment period.

## Discussion and conclusions

Herein, we reported an adult case of extensive burn injury complicated by a generalized vesicular eruption that was suspected of being disseminated zoster due to reactivation of latent VZV. Disseminated zoster during burn treatment is extremely rare [2]. To the best of our knowledge, our case is the second case of reactivation of VZV during the burn treatment. Disseminated zoster that occurs before burn injuries heal severely limits the treatment of burns because of reduced amounts of intact skin for skin grafts. Moreover, VZV infection can involve many organs, including the lungs, heart, liver, and kidneys; and thus in our case it might have accelerated the course of pre-existing multiple MOF that had developed prior to VZV reactivation because of hypotension, inhalation injury, and MDRP infection. In addition, disseminated zoster leads to the problem of nosocomial airborne VZV infection.

VZV is a DNA virus and a member of the human herpes virus (HHV) family, which consists of 8 known types of viruses [3]. Varicella, also known as chickenpox, is the clinical manifestation of the initial VZV infection. Subsequently, VZV remains latent in the trigeminal and dorsal root ganglia. Localized zoster, also known as shingles, is the clinical manifestation of the reactivation of latent VZV [4, 5]. The reactivation of VZV is triggered by a weakened immune system. In most cases, the reactivation of VZV results in shingles, which can occur even in healthy individuals. In shingles, aggregations of vesicles appear unilaterally in a single dermatome or several adjacent dermatomes. The systemic involvement of other organs is rarely seen in shingles. By contrast, in disseminated zoster, a rare manifestation of reactivated latent VZV, the systemic distribution of vesicular eruptions and systemic dissemination of the pathogen to many other organs occurs. Disseminated zoster is rarely seen in immunocompetent patients, but has been reported in immunocompromised patients such as those who have undergone hematopoietic stem cell transplantation and solid organ transplantation, or who have lymphoma or acquired immune deficiency syndrome (AIDS) [6].

Patients with extensive burns have burn-related immunosuppression [7, 8]. However, reports on the reactivation of latent VZV manifesting as either shingles or disseminated zoster in burn patients are rare. The reasons that reactivation of latent VZV is rare in burn patients are unclear [2, 9, 10]. VZV involvement in burn patients is typically seen as varicella in pediatric burn patients who are negative for prior VZV infection or have not been vaccinated [11]. Varicella outbreaks among pediatric burn patients and other patients in the ICU have been reported [12]. Sheridan et al. reported that the incidence of varicella in a pediatric burn unit was 0.1% (12 of 8869 patients) [13]. However, to the best of our knowledge, there have only been 3 reported cases of shingles related to burn treatment (Table 1) [2, 14, 15]. Among those cases only 1 case of shingles occurred before the burn wounds were healed. The patient was an 81-year-old woman with a 12%TBSA burn on her legs who, 46 days after sustaining a burn injury, developed a vesicular exanthem on the intact skin of her right forehead and eyelids, consistent with the dermatome of the first division of the trigeminal nerve. VZV was confirmed by isolation of the virus and elevated titers of complement-fixation antibody [2]. To the best of our knowledge, there are no reports of disseminated zoster occurring during burn treatment.

**Table 1:** Reactivation of varicella zoster virus (VZV) related to burn treatment

Authors	Age (year)	Gender	Immunostatus before burn injury	Extent of Burn (%TBSA^a^)	Onset after burn injury	Site	VZV confirmation	Outcome
Matthews et al., 1979	81	Female	Immunocompetent	12%TBSA	46 days (During burn treatment)	Intact skin of the face (Dermatome of right 1st branch of trigeminal nerve)	Serological, clinical, electron microscopy, and culture	Healed
Lin and Cinat, 2010	29	Male	Immunocompetent	4%TBSA	2.5 years (After burn wound was healed)	Healed skin graft on right ankle	Serological and clinical	Loss of skin graft
Kikuchi and Isa, 1976	70	Female	Immunocompetent	Less than 1%TBSA	3.5 years (After burn wound was healed)	Healed skin graft on right back	Clinical (age, distribution of vesicles on right chest, right back, and right arm.)	Healed
Our case	51	Male	Immunocompetent	60%TBSA	31 days (During burn treatment)	First, intact skin of left knee and left buttock (L4 dermatome). Then, disseminated.	Serological and clinical (age, distribution of primary vesicular exanthem, aggregated style of vesicles, several days of continually erupting rashes, concurrent presence of vesicles, pustules, and crusts.)	Dead

^a^TBSA denotes total body surface area

HSV, a member of the HHV family, is also associated with the occurrence of herpetic lesions in burn patients. HSV includes HSV types 1 (HSV-1) and 2 (HSV-2) and is transmitted by direct contact. Primary infection of HSV or reactivation of latent HSV can result in herpetic skin or mucous membrane lesions. Typically, oral lesions are due to HSV-1 and genital lesions to HSV-2. In burn patients, herpetic lesions associated with HSV are likely to occur in second degree burns and split-thickness skin graft (STSG) harvested sites during the healing process [16–18]. Disseminated herpetic lesions due to HSV are called KVEs and are also known as eczema herpeticum. In the literature, KVEs are more common than latent VZV reactivation in burn patients. A preceding abnormal skin condition is more important as a cause of KVEs than in seen for shingles or disseminated zoster. Therefore, KVE occurs in preceding lesions associated with skin diseases such as atopic dermatitis, psoriasis, contact dermatitis, and burns [19]. Varicella and KVEs in burn patients are likely to involve burned skin in the healing process, which indicates an association between epithelial cell proliferation and viral activity.

Sometimes differentiating between KVEs, varicella, and disseminated zoster is difficult. In our case, a dermatologist examined the patient and diagnosed disseminated zoster 3 days after the onset of the eruptions. The diagnosis of disseminated zoster was made based on the age of the patient, the primary onset of exanthema on the skin of the left L4 dermatome, aggregated pattern of vesicular eruptions, vesicles appearing on normal skin rather than involving healing burn lesions or STSG donor sites, asynchronous generalized eruption of vesicles, and concomitant existence of vesicles, pustules, and crusts. Those findings all support the diagnosis of disseminated zoster instead of varicella or KVE. Serologic testing performed 5 days after the onset of vesicular eruptions also supported the diagnosis of disseminated zoster. However, completely denying the possibility of HSV involvement in our case is difficult, because the simultaneous involvement of HSV and VZV has been reported. Giehl et al. reported that in 1.8% of patients in whom the clinically suspected pathogen was VZV, an additional HSV infection was identified by polymerase chain reaction (PCR) [20]. Although Giehl et al.’s data showed that there are cases of simultaneous involvement of HSV and VZV, they also showed that a clinical diagnosis of zoster, in which suspected pathogen was VZV, based only on clinical manifestations is highly accurate, that was confirmed by PCR. Clinically, there is a need for diagnosing disseminated zoster rapidly in order to prevent nosocomial infection due to potential airborne spread of VZV, whereas HSV in KVEs does not spread by an airborne route.

The first-line treatment of disseminated zoster is acyclovir, which is also true for shingles, varicella, and HSV infection. VZV infection of immunocompromised patients is life-threatening, and early high-dose acyclovir has been reported [21]. In our case, the patient was undergoing 24-h CHDF, which corresponded to 10 mL/min/1.73m^2^ of creatinine clearance [22]. A consensus protocol for acyclovir treatment of patients with renal failure in Japan recommends daily intravenous administration of 250 mg of acyclovir. We administered 500 mg of acyclovir as high-dose acyclovir therapy. Intravenous immunoglobulin has also been reported to be a treatment option [6, 10]. VZV vaccination cannot be used as a preventative in immunocompromised patients, because the VZV vaccine is a live-virus vaccine. A fatal case of disseminated zoster occurring after VZV vaccination of an immunocompromised patient was reported [23].

The molecular trigger of the reactivation of latent VZV is unknown. However, it is clear that deficient cell-mediated immunity in elderly and immunocompromised patients is associated with the reactivation of latent VZV [3]. Immunosuppression is common in patients with extensive burn injuries. The reason why reactivation of latent VZV is rare in burn patients in contrast to the frequent reactivation of HSV remains unclear [2]. Although both VZV and HSV are members of the HHV family and demonstrate similar clinical manifestations, VZV and HSV are different with respect to viral molecular function: e.g., VZV expresses multiple open-reading-frame (ORF) proteins and immediate early proteins during latency, whereas HSV expresses a latency-associated-transcript (LAT) without protein synthesis during latency [3, 24]. Differences between the molecular mechanisms of the reactivation of latent VZV and HSV may be key in accounting for the difference between the incidence rates of disseminated zoster and KVEs in burn patients.

Fever itself is one of the stimulations that promote HSV reactivation. Warren et al. reported that in 46% (190 of 411) of patients undergone artificially induced fever, oral-labial herpes whose cause is thought to be reactivation of HSV occurred, whereas no herpes zoster by reactivation of VZV were observed [25]. Fever commonly seen during burn treatment may be one of the reasons for common HSV reactivation in opposite to rare VZV reactivation.

Nerve growth factor (NGF) and epidermal growth factor (EGF) inhibits HSV reactivation in neuron in vitro [26, 27]. Both NGF and EGF are increasing in skin during healing process after burn injury [28, 29]. We think that the elevated demand for NGF and EGF in the skin after burn injury may lead to decreased levels of NGF and EGF in ganglia, that can promote HSV reactivation in ganglia.

In severely burned patient, the number of CD8+ T cells decreases [30]. Cytolytic CD8+ T cells exist around latent HSV-1 infected ganglia neurons, whereas no cytolytic CD8+ T cells exist around latent VZV infected ganglia neurons [31]. Cytolytic CD8+ T cells are thought to suppress HSV-1 reactivation without causing neural damage. In contrast, cytolytic CD8+ T cells may have no role in suppression of VZV reactivation. HSV-1 presents viral antigen by MHC class I molecules in latently infected neuron, and HSV-1 specific CD8+ T cell interact with HSV-1 latent neuron [32]. On the other hand, VZV downregulates cell surface MHC class I expression. Interaction between latently VZV infected neuron and VZV specific CD8+ T cell may not play a major role in controlling VZV reactivation. Thus, we think that the reduction of CD8+ T cells in severely burn patient may lead to reactivation of HSV but not VZV.

In contrast to the reduction of the number of T cells in severely burn patient, the number of B cells do not alter after burn injury [33]. B cells and antibody production is crucial for preventing some kind of viral reactivation in central nerve system in mice [34]. We think that similar mechanism may exist in VZV latently infected ganglia.

The rate of cytomegalovirus (CMV) infection is considerably high in severely burned patients, that 71% in CMV seropositive burn patients and 13% in CMV seronegative patients [35]. Superinfection with CMV on latent HSV infected human cell provokes HSV reactivation in vitro, whereas VZV reactivation cannot be provoked by CMV superinfection [36]. We think CMV superinfection may play an important role in commonness of HSV reactivation and rarity of VZV reactivation in burn patients.

VZV infects not only neurons but also many other cell types. In severe systemic cases of VZV infection, infected cells can be found in almost every organ, including the spleen, liver, lungs, thymus, digestive tract, kidneys, pancreas, aorta, heart, and brain [1, 3, 6, 37–42]. The VZV organisms in disseminated zoster are thought to be spread through the blood stream [43]. In our case, liver and respiratory dysfunction were evident. However, determining if the dysfunctions were due disseminated zoster or many other burn-related underlying conditions such as MDRP infection, circulatory insufficiency, or systemic inflammation was difficult.

CHDF blood purification combined with PMX-DHP might have been associated with the occurrence of disseminated zoster in our case. CHDF plus PMX-DHP was initiated on day 1 and continued until the day the patient died (day 52). CHDF plus PMX-DHP was used not only to treat renal failure, but also to modulate inflammatory cytokines such as tumor necrosis factor-alpha (TNF-α), interleukin (IL)-1, and IL-6 [22]. Extensive burn injury broadly impairs the functional components of the immune system: e.g., including the function of phagocytes and dendric cells, T-cell homeostasis, immune signaling, and gene expression [7, 8, 44, 45]. In addition, renal failure alone suppresses T-cell functioning and reduces cell-mediated immunity [46]. Although the effects of long-term cytokine modulation by CHDF plus PMX-DHP in patients with extensive burn injuries are unknown, these interventions might activate VZV by an unknown mechanism.

The prevention of airborne transmission of VZV to other patients and health care workers is clinically important. The reported incidence rate of disseminated zoster is lower than the incidence rate of KVE in burn patients. However, there is a possibility that disseminated zoster in burn patients is overlooked, because disseminated zoster has been rarely known to occur as a complication in burn patients. Varicella outbreaks among pediatric burn patients and other ICU patients have been reported [11, 12]. Anugulruengkitt et al. reported that the prevalence of VZV non-immune healthcare workers was 2.6% [47]. In our hospital, all healthcare workers are checked for the presence of anti-VZV IgG antibody when they start working, and the results are stored in an online database. Associated with our case, no healthcare workers without immunity to VZV were identified. Patients who were in the ICU during the period spanning the eruption of vesicles on day 31 to when the patient was transferred to the negative-pressure isolation room were tested for anti-VZV IgG antibody. Fortunately, patients without immunity to VZV were not found.

In conclusion, we treated a severely burned patient with suspected disseminated zoster basing on his clinical findings and results of serology. Immediate isolation of the patient in a negative-pressure room was implemented to prevent airborne infections of VZV. Burn-related immunosuppression and long-term cytokine modulation by CHDF plus PMX-DHP might lead to rare, fatal reactivation of latent VZV infection.

## Abbreviations

AIDS: Acquired immunodeficiency syndrome
CHDF: Continuous hemodialysis and filtration
ELISA: Enzyme-linked immunosorbent assay
HHV: Human herpes virus
HSV: Herpes simplex virus
Ig: Immunoglobulin
IL: Interleukin
KVE: Kaposi’s varicelliform eruption
L4: 4th lumbar
LAT: Latency-associated-transcript
MDRP: Multiple-drug-resistant *Pseudomonas aeruginosa*
MOF: Multiple organ failure
ORF: Open-reading-frame
PCR: Polymerase chain reaction
PMX-DHP: Polymyxin B-immobilized fiber column direct hemoperfusion
ROSC: Return of spontaneous circulation
STSG: Split-thickness skin graft
TBSA: Total body surface area
TNF-α: Tumor necrosis factor-alpha
VZV: Varicella zoster virus
